# De Novo Synthesis of Resveratrol from Sucrose by Metabolically Engineered *Yarrowia lipolytica*

**DOI:** 10.3390/biom14060712

**Published:** 2024-06-16

**Authors:** Gehad G. Ibrahim, Madhavi Perera, Saadiah A. Abdulmalek, Jinyong Yan, Yunjun Yan

**Affiliations:** 1 Key Laboratory of Molecular Biophysics of the Ministry of Education, College of Life Science and Technology, Huazhong University of Science and Technology, Wuhan 430074, China; ggibrahim@zu.edu.eg (G.G.I.); wwkawshalya@gmail.com (M.P.); 2 Department of Genetics, Faculty of Agriculture, Zagazig University, Zagazig 7120001, Egypt; 3 Department of Electrical, Electronic and Telecommunication, Faculty of Engineering, General Sir John Kotelawala Defence University, Rathmalana 10390, Sri Lanka; 4 Department of Biology, Faculty of Science, Sana’a University, Sana’a 1247, Yemen; micro.saadiah2015@hotmail.com

**Keywords:** resveratrol, microbial cell factory, metabolic engineering, *Yarrowia lipolytica*

## Abstract

Resveratrol, a phenylpropanoid compound, exhibits diverse pharmacological properties, making it a valuable candidate for health and disease management. However, the demand for resveratrol exceeds the capacity of plant extraction methods, necessitating alternative production strategies. Microbial synthesis offers several advantages over plant-based approaches and presents a promising alternative. *Yarrowia lipolytica* stands out among microbial hosts due to its safe nature, abundant acetyl-CoA and malonyl-CoA availability, and robust pentose phosphate pathway. This study aimed to engineer *Y. lipolytica* for resveratrol production. The resveratrol biosynthetic pathway was integrated into *Y. lipolytica* by adding genes encoding tyrosine ammonia lyase from *Rhodotorula glutinis*, 4-coumarate CoA ligase from *Nicotiana tabacum*, and stilbene synthase from *Vitis vinifera*. This resulted in the production of 14.3 mg/L resveratrol. A combination of endogenous and exogenous malonyl-CoA biosynthetic modules was introduced to enhance malonyl-CoA availability. This included genes encoding acetyl-CoA carboxylase 2 from *Arabidopsis thaliana*, malonyl-CoA synthase, and a malonate transporter protein from *Bradyrhizobium diazoefficiens*. These strategies increased resveratrol production to 51.8 mg/L. The further optimization of fermentation conditions and the utilization of sucrose as an effective carbon source in YP media enhanced the resveratrol concentration to 141 mg/L in flask fermentation. By combining these strategies, we achieved a titer of 400 mg/L resveratrol in a controlled fed-batch bioreactor. These findings demonstrate the efficacy of *Y. lipolytica* as a platform for the de novo production of resveratrol and highlight the importance of metabolic engineering, enhancing malonyl-CoA availability, and media optimization for improved resveratrol production.

## 1. Introduction

Resveratrol (3,4′,5-trihydroxystilbene) is a plant-derived stilbenoid with extensive pharmacological potential [1]. It exhibits anticancer, anti-inflammatory, antioxidant, antibacterial, antiviral, anti-aging, and neuroprotective properties. Additionally, it helps manage neurodegenerative disorders, enhancing bone and ocular health, and reducing diabetic complications [2,3,4,5,6,7]. Despite clinical limitations, its cardiovascular benefits have been established in preclinical models [8,9]. Additionally, it is used in topical skincare products [10,11,12,13,14], and can potentially treat viral infections such as COVID-19 [15]. Reflecting its wide array of health benefits, the global market for resveratrol was valued at USD 71.9 million in 2020 and is expected to reach USD 131.0 million by 2030, with a compound annual growth rate (CAGR) of 6.2% from 2021 to 2030 [16]. This growth highlights the need for enhanced production methods.

**Figure 1:**
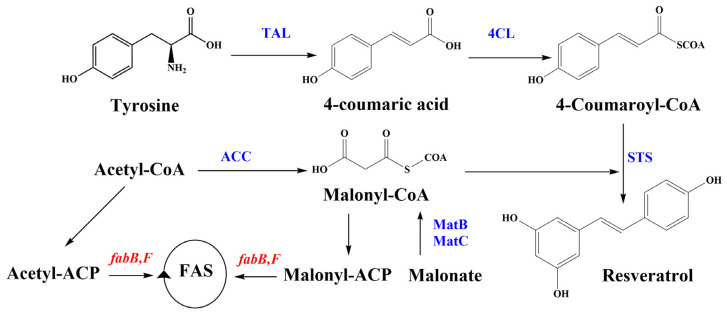
Resveratrol synthesis pathway and genes and enzymes involved in *Y. lipolytica* engineering in this study. Genes encoding enzymes in blue were inserted into the yeast. Enzymes encoded by red genes were inhibited using the antibiotic cerulenin. 4CL, 4-coumaroyl-coA ligase; ACC, acetyl-CoA carboxylase; Acetyl-CoA, acetyl-coenzyme A; *fabB/fabF/fabH*, genes that encode the 3-ketoacyl-ACP synthase I/II; MatB, malonyl-CoA synthetase; MatC, malonate carrier protein; STS, stilbene synthase; TAL, tyrosine ammonia-lyase.

Resveratrol biosynthesis primarily utilizes malonyl-CoA, phenylalanine, or tyrosine as substrates. The pathway begins with the conversion of tyrosine to 4-coumaric acid (*p*-CA) via tyrosine ammonia-lyase (TAL) or phenylalanine to cinnamic acid via phenylalanine ammonia-lyase (PAL), which is then converted to *p*-CA by cinnamate-4-hydroxylase (C4H). *p*-CA is then converted to 4-coumaroyl-CoA by 4-coumaroyl coenzyme A ligase (4CL). Finally, stilbene synthase (STS) catalyzes the condensation of *p*-coumaroyl-CoA with three malonyl-CoA molecules to produce resveratrol [11,17]. The essential enzymatic reactions for resveratrol production through the tyrosine pathway and the metabolic engineering strategies used in this study are illustrated in Figure 1.

Microbial synthesis offers a viable alternative to plant-based methods, providing advantages such as faster production, lower costs, and easier genetic manipulation [18,19]. *Yarrowia lipolytica*, known for its Generally Recognized As Safe (GRAS) status and heterologous gene expression capabilities [20], is an ideal host for resveratrol production due to its abundant acetyl-CoA and malonyl-CoA supply and efficient pentose phosphate (PP) pathway [21,22] making. However, malonyl-CoA consumption in fatty acid biosynthesis necessitates strategies to enhance its availability. This can be achieved by inhibiting fatty acid synthesis or increasing the intracellular concentration of malonyl-CoA. Enhancing the malonyl-CoA precursor can also be attained through several methods, including the overexpression of the acetyl-CoA carboxylase gene (*ACC*) [23] or using a module that relies on external supplementation [24,25]. This research reports the metabolic engineering of *Y. lipolytica* for resveratrol bioproduction and documents the attempt to increase the malonyl-CoA intracellular concentration with the *ACC2*, *matBC* pathway and cerulein approaches, which have not been previously reported in this yeast.

## 2. Materials and Methods

### 2.1. Chemicals

Yeast extract, tryptone, and peptone were sourced from Thermo Fisher Scientific Inc., Waltham, MA, USA (LP0021T, LP0024B, and LP0037B, respectively). NaCl, sucrose, and glucose were obtained from Sinopharm Chemical Reagent Co., Shanghai, China (100193088, 100214190, and 10010518, respectively). Ampicillin, kanamycin, and biotin were acquired from Solarbio, Beijing, China (K8180, K8020, and D8150, respectively). Yeast nitrogen base was purchased from Biosharp, Hefei, China (BS905). Leucine and agar were sourced from BioFroxx, Saiguo Biotechnology Co., Ltd., Guangzhou, China (1215GR025 and 8211KG001, respectively). Cerulenin was obtained from Yuan Ye, Shanghai, China (S43646). Resveratrol was obtained from Macklin, China, Shanghai (R817263). Malonate was purchased from Sigma-Aldrich, Darmstadt, Germany (792535). *p*-CA was purchased from Aladdin Bio-Chem Technology Co., Shanghai, China (C108514).

### 2.2. Strains and Culture Conditions

One Shot™ TOP10 Chemically Competent *E. coli* (C4040-03, Thermo Fisher Scientific Inc., USA) was used for plasmid construction and amplification. The *Y. lipolytica* strain Po1f (Donated by Madzak C., UMR 782 SayFood, INRAE, Paris, France) was used as the host for recombinant plasmid transformation and expression [26]. Bacterial cells were cultured in Lysogeny Broth (LB) medium (0.5% yeast extract, 1% tryptone, and 1% NaCl, pH 7.0) at 37 °C, 200 rpm with 100 mg/L ampicillin or 50 mg/L kanamycin for antibiotic selection. *Y. lipolytica* was cultured in Yeast Extract Peptone (YP) medium (1% yeast extract and 2% peptone) or Yeast Nitrogen Base (YNB) medium (0.67% yeast nitrogen base, 4 × 10^−5^% biotin, with/without 0.005% leucine) with 2%, 5%, or 8% glucose/sucrose at 28 °C, 200 rpm. Supplements of 2 mM *p*-CA, 1 mg/L cerulenin, or 20 mM malonate were added as needed. A YP medium with 2% glucose was considered as Yeast Extract Peptone Dextrose (YPD) medium. Solid media were prepared by adding 2% agar. Strains were cryopreserved in 25% glycerol after growth in 2X YPD medium.

### 2.3. Genes, Plasmid, and Strain Construction

The integrative plasmid pINA1312 [26] was utilized to express the heterologous genes encoding tyrosine ammonia lyase (RgTAL), 4-coumarate CoA ligase (Nt4CL), and stilbene synthase (VvSTS). In contrast, the integrative plasmid pINA1269 [27] was used to express heterologous genes encoding acetyl-CoA carboxylase (AtACC2), malonyl CoA synthase (BdMatB), and malonate transporter protein (BdMatC). Genes were codon-optimized according to *Y. lipolytica* codon usage and synthesized by GeneScript (Piscataway, NJ, USA). Plasmids were transformed into One Shot™ TOP10 Chemically Competent *E. coli*, according to the manufacturer’s guidelines. *Y. lipolytica* transformation was performed by a modified lithium acetate method adapted from Chen et al. [28]. All transformants were verified by PCR. Extraction of plasmid and yeast genomic DNA, along with gel extraction and purification of DNA fragments, were carried out using kits provided by Omega Biotech Co., Ltd. (Shanghai, China). All primers were synthesized by Tsingke Biotech Co., Ltd. (Beijing, China). Detailed information regarding the synthesized gene sequences, strains, plasmids, and primers are provided in Supplementary Materials: Tables S1 and S2.

### 2.4. Small-Scale Fermentation

All small-scale fermentations were conducted in 500 mL flasks. The glycerol stock of the target strain was streaked on YNB agar plates supplemented with 20 g/L glucose to isolate single colonies. These colonies were then cultured in 10 mL of the same medium in 50 mL tubes for 24 h at 28 °C and 200 rpm. Appropriate seed culture volumes were then used to inoculate 50 mL YPD media, achieving an initial optical density at 600 nm (OD_600_) of 0.1–0.5, and incubated at 28 °C and 200 rpm for 120 h. Malonate was added to the media to analyze the expression of *matB* and *matC* genes.

To examine the precursor availability for resveratrol synthesis, YNB media with 20 g/L glucose containing *p*-CA and/or cerulenin was used. For media optimization, YP and YNB media supplemented with different carbon sources at various concentrations were used. Samples were collected at predetermined time points throughout the experiment and analyzed by High-Performance Liquid Chromatography (HPLC). All experimental data were obtained in biological triplicates. 

### 2.5. Fed-Batch Fermentation

The target strain’s glycerol stock was utilized to obtain a single colony, which was then used to inoculate 10 mL YNB medium for 24 h at 28 °C with shaking at 200 rpm. This culture was used to inoculate 120 mL YNB medium in a 500 mL flask under the same conditions for another 24 h. Subsequently, the final culture was used to inoculate a 3 L bioreactor (Baoxing Bio-Engineering, Shanghai, China) with an initial OD_600_ of 0.5. The bioreactor was equipped with continuous monitoring probes for dissolved oxygen (DO), pH, and temperature and an automated system for nutrient addition.

Fermentation proceeded at 28 °C over ten days, starting with 1.2 L of YP medium containing 50 g/L sucrose. The pH was maintained at 6.0 by the automated addition of 40% (*v*/*v*) KOH or 10% (*v*/*v*) HCl as needed. Dissolved oxygen levels were regulated through controlled agitation and aeration. Upon depletion of the initial sucrose, a 5-fold concentrated feeding medium containing 500 g/L sucrose and 20 mM malonate was incrementally added. Samples were routinely collected every 12 h to measure OD_600_, *p*-CA, and resveratrol concentration. All measurements were performed in triplicates.

### 2.6. Analytical Methods

To determine *p*-CA and resveratrol concentrations, each sample was mixed with an equal amount of absolute ethanol, vortexed vigorously, and then centrifuged at 12,000 rpm for 10 min. The supernatant was subsequently filtered through a 0.22 μm filter membrane and stored at −20 °C until further analysis. HPLC analyses were performed using e an LC 5090 (FULI INSTRUMENT) equipped with an Accucore aQ C18 column (100 mm × 2.1 mm, with particle size 2.6 μm, Thermo Scientific, Waltham, MA, USA) and a variable-wavelength detector. The analytical conditions were as follows: the column oven temperature was maintained at 25 °C, the flow rate was set to 0.3 mL/min, and 20 μL of each sample was injected for quantification. The mobile phase consisted of solvent A (acetonitrile) and solvent B (0.1% formic acid in water). The elution started with 5% solvent A for 2 min, followed by a linear gradient to 70% solvent A over 8 min, maintained for 5 min, and then restored to the initial conditions over 5 min. The conditions remained constant until the end of the run, resulting in a total run time of 25 min. Both *p*-CA and resveratrol were detected at 306 nm, with retention times of approximately 10.5 and 12.6 min, respectively. Standard curves for each compound were generated using reference standards of ≥99% purity for resveratrol and ≥98% purity for *p*-CA.

Cell densities were monitored by measuring the OD_600_ of the cultures using an Infinite 200 Pro microplate reader after appropriate dilution (2–20 fold). Concentrations of glucose and sucrose in the culture medium were estimated using Glucose/Sucrose Assay Kits supplied by Beijing Solarbio Science and Technology Co., Beijing, China. 

### 2.7. Statistical Analysis

All results were presented as mean values ± standard deviation (SD) from at least three independent experiments. The statistical significance of differences between data sets was determined using one-way analysis of variance (ANOVA) followed by Tukey’s multiple comparison test. A *p*-value of less than 0.05 was considered statistically significant. Data visualizations were created with OriginPro software, version 2024 (OriginLab Corporation, Northampton, MA, USA).

## 3. Results and Discussion

### 3.1. De Novo Production of Resveratrol

**Figure 2:**
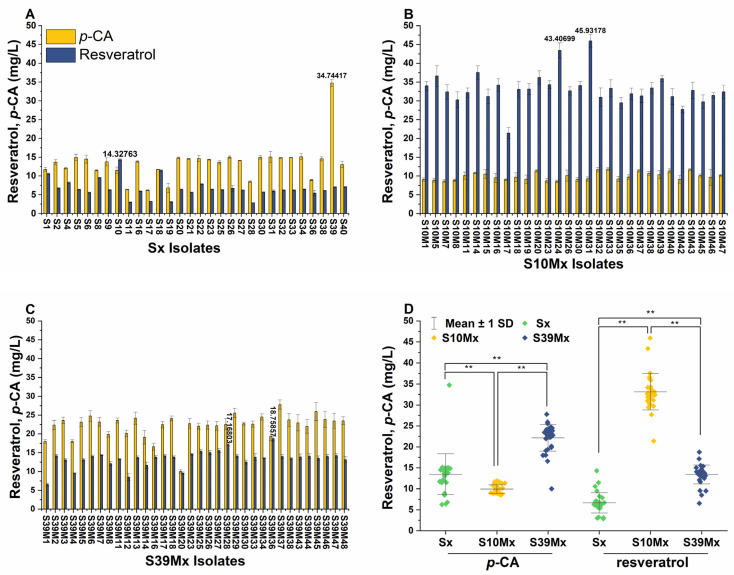
*p*-CA and resveratrol production in different engineered *Y. lipolytica* strains after 120 h of cultivation in YPD media. (**A**) *p*-CA and resveratrol production in the Sx group strains, derived from the *Y. lipolytica* Po1f strain and recombinant with the pINA1312-ST4C cassette. (**B**) *p*-CA and resveratrol production in the S10x group strains, derived from the S10 strain and recombinant with the pINA1312-ST4C and pINA1269-BCA cassettes. (**C**) *p*-CA and resveratrol production in the S39x group strains, derived from the S39 strain and recombinant with the pINA1312-ST4C and pINA1269-BCA cassettes. (**D**) Comparison of *p*-CA and resveratrol average concentrations obtained from the three groups. ** indicate significant differences at a 0.01 significance level.

The de novo production of resveratrol in the *Y. lipolytica* Po1f strain was successfully achieved by introducing *TAL* from *Rhodotorula glutinis*, *4CL* from *Nicotiana tabacum*, and *STS* from *Vitis vinifera*. These genes were combined into a single open reading frame, linked by a Thosea asigna virus 2A (T2A) self-cleaving peptide and co-expressed under the synthetic hp4d promoter within the pINA1312-ST4C plasmid (the plasmid construction is described in Figure S1). The T2A peptide strategy provides a rapid and straightforward method to integrate the entire resveratrol biosynthetic pathway into a single vector. This approach eliminates the need for a large vector and ensures the stoichiometric expression of the pathway genes [29]. In *Y. lipolytica*, non-homologous end joining (NHEJ) is the primary genome repair mechanism, which represents challenges for precise gene integration [30,31]. This often results in the random insertion of genes, causing variability in genomic integration sites and expression levels [32], which require screening multiple transformants. Transforming the *Y. lipolytica* Po1f strain with the pINA1312-ST4C plasmid resulted in 30 resveratrol-producing strains (Sx group). Among this group, strain S10 exhibited the highest resveratrol titer, yielding 14.3 ± 0.3 mg/L (Figure 2A). The confirming PCR and HPLC results are provided in the Supplementary Materials, Figures S2–S5.

Interestingly, except for strain S10, most strains produced higher levels of *p*-CA than resveratrol. This observation is consistent with prior studies reporting that *Y. lipolytica* can effectively produce considerable amounts of *p*-CA in the YPD medium [33,34]. Notably, strain S39 produced the highest titer of *p*-CA, reaching 34.7 ± 0.9 mg/L, as illustrated in Figure 2A. These high *p*-CA concentrations underscore a potential metabolic bottleneck in converting *p*-CA to resveratrol, likely due to insufficient intracellular malonyl-CoA availability, a common limitation in the production of phenylpropanoids [33,35].

### 3.2. Enhancing Malonyl-CoA Availability

Malonyl-CoA is an essential substrate in resveratrol biosynthesis, as each resveratrol molecule is synthesized via the STS enzyme that requires three malonyl-CoA units. However, the primary metabolic pathway for malonyl-CoA is directed towards fatty acid synthesis, which limits its availability for resveratrol production [2]. To increase the malonyl-CoA availability and evaluate its impact on resveratrol synthesis, we employed a dual strategy. This involved utilizing ACC2, an enzyme responsible for converting acetyl-CoA into malonyl-CoA, and the alternative malonate pathway, including MatB and MatC enzymes. The *ACC2* gene from *Arabidopsis thaliana*, and *matB* and *matC* genes from *Bradyrhizobium diazoefficiens* were assembled into the pINA1269 vector as a single operon, separated by T2A peptides, and regulated by the growth-phase-dependent hp4d promoter (the plasmid construction is described in Figure S6). Strains S10 and S39 were further engineered to harbor the pINA1269-ACC-BCA construct. The confirming PCR results are available in the Supplementary Materials, Figures S7 and S8.

From each strain, S10 and S39, 30 resveratrol-producing strains were derived, named the S10Mx and S39Mx groups, respectively. The subsequent screening of these strains revealed that strain S10M31 produced 45.9 ± 1.7 mg/L resveratrol, a 3.2-fold increase over the highest resveratrol-producing strain from an earlier selection (Figure 2B). This improvement in resveratrol titers was consistent across both groups (Figure 2D), indicating the success of the *ACC2* approach. Notably, the S10Mx group outperformed the S39Mx group in resveratrol production by 2.5-fold despite displaying 2.3 times lower *p*-CA levels, suggesting that the genomic integration in different sites may influence the gene expression in the integrated cassette. Decoene and Schuetze and Meyer found that in *S. cerevisiae* and *Aspergillus niger*, the efficiency of 2A peptides within tri-cistronic constructs is position-dependent, with the lowest activity at the first position [36,37]. Given that the *STS* gene occupies the first gene position in the pINA1312-ST4C plasmid, it is hypothesized that varying integration sites might impact this initial position within the integrated cassette, leading to reduced *STS* gene expression in strains derived from S39 compared to those from S10. This expression variation could potentially explain the lower final resveratrol titer and the accumulation of *p*-CA in the S39Mx group.

The strategy of overexpressing *ACC1* to increase the cytosolic malonyl-CoA and, consequently, resveratrol production has been applied to various microorganisms, including *S. cerevisiae*, *Y. lipolytica*, *E. coli*, and *E. coli–S. cerevisiae* co-cultures, either alone or in combination with other metabolic engineering strategies [38,39,40]. In this study, the paralog gene *ACC2* was also found to be effective in increasing the malonyl-CoA levels. However, this approach is limited by the cytoplasmic availability of precursors. Therefore, a second approach involving supplementation with external substrates was implemented.

### 3.3. Expression of matB and matC Genes

To further enhance malonyl-CoA availability, we implemented another module involving the heterologous expression of the genes encoding MatC and MatB. This approach includes adding malonate, an affordable and readily available substance, to the fermentation process. MatC facilitates the entry of malonate into the cell, while MatB converts malonate into malonyl-CoA.

**Figure 3:**
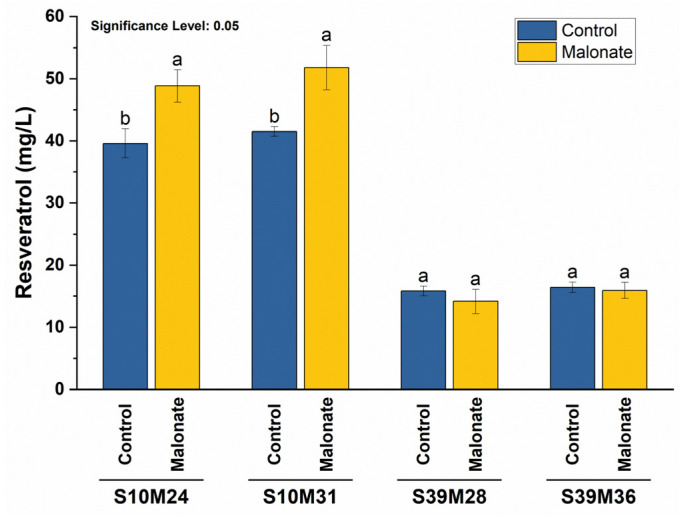
The effect of malonate on resveratrol production in different strains. To increase malonyl-CoA levels through the expression of the *matB* and *matC* genes, 20 mM malonate was added to the YPD media. Resveratrol concentrations were estimated after 120 h of cultivation. Control fermentations lacked malonate. Letters represent the significance of differences. Distinct letters indicate significant differences at a 0.05 significance level.

Four strains, S10M24, S10M31, S39M28, and S39M36, the highest resveratrol producers in their respective groups, were used to assess this strategy. The resveratrol concentrations in the S10M24 and S10M31 (S10Mx) strains exhibited a significant increase, reaching 48.8 ± 2.6 and 51.8 ± 3.6 mg/L resveratrol, respectively, representing a 1.23-fold enhancement over the control groups (Figure 3). However, the S39Mx strains did not show significant changes in the resveratrol concentration, and the *p*-CA levels remained consistent across all strains. The application of the *matBC* pathway strategy successfully elevated resveratrol production in this study.

In several studies, the heterologous expression of the *matB* and *matC* genes has been selected as the primary strategy for boosting malonyl-CoA levels in *E. coli* [41,42]. Furthermore, employing *matB* and *matC* genes from *R. trifolii* has been previously demonstrated to enhance resveratrol production in *E. coli* [24,25]. To our knowledge, this is the first use of this combined metabolic engineering strategy to upgrade the malonyl-CoA level in *Y. lipolytica*.

### 3.4. The Effects of Cerulenin on Resveratrol Production

To further elucidate the precursor availability for resveratrol production, we considered the competition for malonyl-CoA between resveratrol biosynthesis and lipid formation in this oleaginous yeast [43]. The direct knockout of *fab* genes, which is involved in fatty acid synthesis, is often lethal, necessitating alternative approaches to reduce malonyl-CoA consumption by lipid biosynthesis. One such method involves the utilization of cerulenin, a known inhibitor of β-ketoacyl-acyl carrier protein synthases (KAS) I and II encoded by the *fabB* and *fabF* genes. Cerulenin has been demonstrated to effectively suppress malonyl-CoA consumption in fatty acid synthesis [23]. 

**Figure 4:**
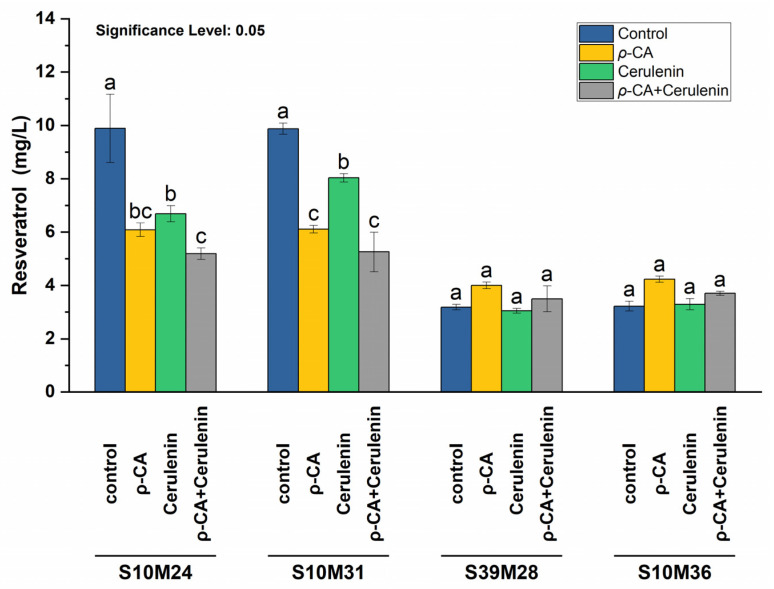
The effect of *p*-CA and cerulenin on resveratrol production in different strains. Either 2 mM *p*-CA, 1 mg/L cerulenin, or both were added to YNB media. Resveratrol concentrations were estimated after 120 h of cultivation. Control fermentations lacked *p*-CA and cerulenin. Letters represent the significance of differences. Distinct letters indicate significant differences at a 0.05 significance level.

Cerulenin was applied alone or in combination with *p*-CA to the S10M24, S10M31, S39M28, and S39M36 strains. These strains exhibited distinct profiles in resveratrol and *p*-CA production. Under all conditions, the resveratrol levels were consistently higher in the S10M24 and S10M31 strains, supporting our hypothesis that integration sites influence gene expression in different strains. Notably, the S10Mx strains showed significantly higher resveratrol levels without cerulenin and *p*-CA supplementation (Figure 4). To control *p*-CA, a YNB medium was used. A comparison of S39Mx strains grown in YPD and YNB revealed the significant impact of the medium on *p*-CA production (Figure S9). However, neither the addition of *p*-CA alone nor in combination with cerulenin significantly boosted resveratrol production in these strains when grown in YNB. Previous studies have highlighted cerulenin’s capability to increase malonyl-CoA availability and resveratrol production in bacteria such as *E. coli* and *Corynebacterium glutamicum* [44,45]. Nevertheless, our yeast model did not respond similarly, which is consistent with findings in *Rhodotorula toruloides*, another oleaginous yeast [43]. 

Collectively, these findings suggest that the endogenous levels of *p*-CA and malonyl-CoA in the engineered strains might be sufficient to sustain resveratrol synthesis under the given conditions. Additionally, they highlighted the critical role of the culture medium composition in the biosynthesis of resveratrol and its precursors.

### 3.5. Media Optimization for Resveratrol Production

In *Y. lipolytica*, the choice of medium significantly influences metabolite production, including resveratrol [33,46,47]. This yeast exhibits metabolic versatility, enabling it to utilize diverse carbon sources. This flexibility can be enhanced through genetic manipulation to use novel carbon sources, such as engineering the Po1f strain to utilize sucrose [48]. The S10M31 strain was used to determine the optimal medium for resveratrol production. Fermentations were conducted in 500 mL flasks containing 100 mL of YP or YNB media. The media were supplemented with different concentrations of glucose or sucrose (20, 50, or 80 g/L) and labeled accordingly. Microbial growth, as well as *p*-CA and resveratrol concentrations, were monitored every 24 h. 

Consistent with previous findings, the YP media displayed higher levels of resveratrol production compared to YNB across all tested parameters. Notably, the YNB media showed a significant decline in pH (Figure S10), which is known to negatively impact microbial growth, physiology, carbon source utilization, and subsequent resveratrol production, as observed earlier by Sáez-Sáez et al. [33].

**Figure 5:**
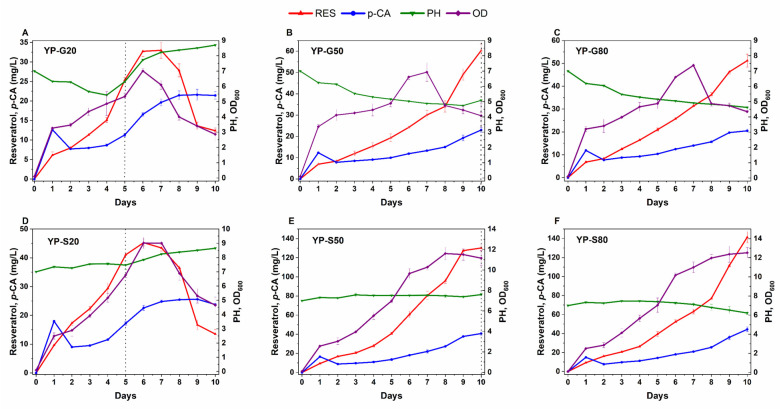
The effect of carbon source and its concentration on resveratrol and *p*-CA production by the S10M31 strain. The strain was fermented for 10 days in YP media supplemented with 20, 50, or 80 g/L of either glucose (G) or sucrose (S) as the carbon source. Resveratrol, *p*-CA, pH, and OD_600_ were measured every 24 h. Panels (**A**–**C**) show the results for glucose concentrations of 20, 50, and 80 g/L, respectively, while panels (**D**–**F**) show the results for sucrose concentrations of 20, 50, and 80 g/L, respectively. Dotted lines indicate the depletion of the carbon source.

Sucrose emerged as an effective carbon source for resveratrol production across various media types and concentrations (Figure 5). Likewise, YP sucrose media exhibited higher microbial growth and *p*-CA concentrations. Remarkably, resveratrol synthesis continued up to the 10th day of fermentation in the YP media with sucrose, reaching concentrations of 130.2 ± 3.2 mL/L and 141.4 ± 5.9 mg/L at 50 g/L and 80 g/L sucrose concentrations, respectively (Figure 5E,F). Despite cells reaching the death phase, resveratrol levels continued increasing in the YP-S50 and YP-S80 media, likely due to the growth-phase-dependent hp4d promoter, which enabled sustained resveratrol synthesis in the presence of a sufficient carbon source. However, the limited catalytic efficiency of biosynthetic enzymes may constrain further resveratrol production.

*Y. lipolytica* growth typically stops upon complete carbon source consumption, followed by increased acid utilization, which indicates the end of the culture [49]. This pattern likely explains the pH increase in the YP-G20 and YP-S20 cultures after carbon source depletion, which was followed by a decline in growth and resveratrol concentrations.

To investigate the observed decline in resveratrol concentrations in the YP-G20 and YP-S20 cultures, a degradation assay using the S10M31 strain was conducted. In this assay, 100 mg/L resveratrol was used as the sole carbon source to replace the glucose in YPD media, followed by 120 h fermentation as described by Zhang et al. [43]. The results revealed the strain’s inability to utilize resveratrol as a carbon source (Figure S11), suggesting that the reduction in resveratrol levels may be due to oxidation and natural instability in alkaline conditions [50] rather than microbial consumption.

Multiple studies underscore the critical role of carbon sources and their concentrations in modulating resveratrol production. For instance, doubling the glucose concentration led to a 3-fold increase in resveratrol titer produced by *C. glutamicum* [45]; a similar pattern was observed in *Y. lipolytica* [33]. Additionally, variations in the resveratrol yield obtained from *Scheffersomyces stipitis* highlighted the substantial influence of the media composition, carbon source type, and carbon source concentration on its biosynthesis and accumulation [51].

### 3.6. Fed-Batch Fermentation

To evaluate the *Y. lipolytica* S10M31 strain for laboratory-scale resveratrol production, successful strategies were combined with a fed-batch fermentation in a 3 L bioreactor. The fermentation was initiated in YP medium containing 50 g/L sucrose. Once the initial carbon source was depleted, continuous feeding of the supplemented medium was carried out at a constant rate, adding a total of 450 g sucrose over 10 days. 

**Figure 6:**
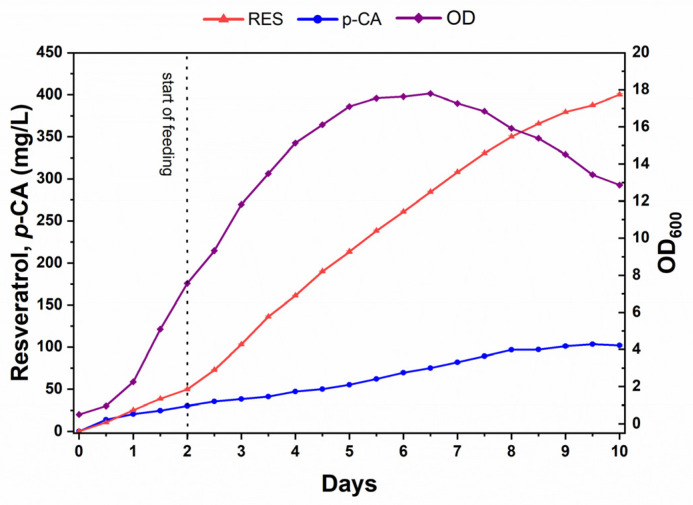
Fed-batch fermentation of the S10M31 strain in a 3L bioreactor. The strain was grown at 28 °C in YP medium supplemented with sucrose and malonate. Fermentation was carried out for 10 days. Resveratrol, *p*-CA, and OD_600_ were measured every 12 h. The dotted line indicates the time when feeding was started.

Resveratrol concentrations continued to increase until day 10, reaching 400.1 ± 1 mg/L (Figure 6). Prior studies on *Y. lipolytica* for resveratrol synthesis have reported a wide range of yields [52], from a few milligrams [53] per liter up to 22.5 g/L, the highest titer obtained reported by Liu et al. [31] (Table S3). Although the titers observed in this study are moderate, we succeeded in the de novo synthesis of resveratrol using cost-effective carbon sources and applied an effective method to augment malonyl-CoA availability.

## 4. Conclusions

*Y. lipolytica* was successfully engineered with resveratrol pathway genes *RgTAL*, *Nt4CL*, and *VvSTS*. Two metabolic engineering models for increasing malonyl-CoA were implemented using the combination of the *AtACC2*, *BdmatB*, and *BdmatC* genes. After optimizing the growth conditions, a resveratrol yield of 400 mg/L was obtained from fed-batch fermentation. This study represents a metabolic engineering step towards utilizing *Y. lipolytica* as an effective platform for the de novo production of resveratrol. 

## Data Availability

Data are contained within the article and Supplementary Materials.

## Supplementary Materials

The following supporting information can be downloaded at: https://www.mdpi.com/article/10.3390/biom14060712/s1, Figure S1: Schematic diagram of pINA1312-ST4C recombinant plasmid with *VvSTS*, *RgTAL*, and *Nt4CL* genes, linked by T2A linker. Figure S2: Colony PCR results of *E. coli* TOP10 recombinants with pINA1312-ST4C plasmid. Figure S3: PCR results of *Y. lipolytica* Po1f recombinant strains with pINA1312-ST4C plasmid. Figure S4: HPLC chromatographs for the *p*-CA and resveratrol peaks in selected samples. Figure S5: The HPLC chromatograph for *p*-CA and resveratrol standards. Figure S6: Schematic diagram of pINA1269-BCA recombinant plasmid containing *BdmatB*, *BdmatC*, and *AtACC2* genes, linked by T2A linker. Figure S7: Colony PCR results of *E. coli* TOP10 recombinants with pINA1269-BCA plasmid. Figure S8: PCR confirmation results of transformant strains originating from S10. Figure S9: The effect of adding *p*-CA and cerulenin to the YNB media on *p*-CA production in different strains after 120 h culturing in YNB media. Figure S10: The effect of carbon source and its concentration on resveratrol production by S10M31 strain. Figure S11: Degradation assays for resveratrol in S10M31 strain; Table S1: Strain and plasmids used in this work; Table S2: Primers used in this work; Table S3: The highest resveratrol yield among *Yarrowia lipolytica* and their engineering strategies, DNA sequences used in this work.
