# Supplementary material for: De Novo Synthesis of Resveratrol from Sucrose by Metabolically Engineered Yarrowia lipolytica
Source: Biomolecules. 2024 Jun 16;14(6):712. doi: 10.3390/biom14060712 (PMC11201955; doi:10.3390/biom14060712)
Supplement: Supplementary file 1 [file biomolecules-14-00712-s001.zip › biomolecules-2999018-supplementary.pdf]

Supplementary Materials

De novo synthesis of resveratrol from sucrose by metabolically engineered *Yarrowia lipolytica*

Gehad G. Ibrahim[a,b], Madhavi Perera[a,c], Saadiah A. Abdulmalek[d], Jinyong Yan[a*], Yunjun Yan[a*]

[a] Key Laboratory of Molecular Biophysics of the Ministry of Education, College of Life Science and Technology, Huazhong University of Science and Technology, Wuhan 430074, P. R. China.

[b] Department of Genetics, Faculty of Agriculture, Zagazig University, Zagazig 7120001, Egypt.

[c] Department of Electrical, Electronic and Telecommunication, Faculty of Engineering, General Sir john Kotelawala Defence University, Rathmalana 10390, Sri Lanka.

[d] Department of Biology, Faculty of Science, Sana'a University, Sana'a 1224, Yemen.

*Corresponding author: Jinyong Yan, E-mail address: yjiny@126.com; Yunjun Yan, E-mail address: yanyunjun@hust.edu.cn

Table S1. Strain and plasmids used in this work

Strain/Plasmid	Relevant genotype	Origin
E. coli Top10	One Shot™ TOP10 Chemically Competent *E. coli,* Catalog Number C4040-03<br>For plasmid construction and maintenance	Thermo Fisher Scientific Inc., USA
Y. lipolytica Po1f	MatA*, leu2-270, ura3-302, xpr2-322, axp-2*<br>For transformation – parental strain	[1]
S10	Po1f, *RhTAL- Nt4CL-VvSTS*	This study
S39	Po1f, *RhTAL-Nt4CL-VvSTS*	This study
S10M24	Po1f, *RhTAL-Nt4CL-VvSTS-AtACC2-BdMatB-BdMatC*	This study
S10M31	Po1f, *RhTAL-Nt4CL-VvSTS-AtACC2-BdMatB-BdMatC*	This study
S39M28	Po1f, *RhTAL-Nt4CL-VvSTS-AtACC2-BdMatB-BdMatC*	This study
S39M36	Po1f, *RhTAL-Nt4CL-VvSTS-AtACC2-BdMatB-BdMatC*	This study
pINA1312	Template plasmid - Kan^R	[2]
pINA1269	Template plasmid - Amp^R	[1]
pINA1312-ST4C	pINA1312- *RhTAL-Nt4CL-VvSTS*	This study
pINA1269-ACC-BCA	pINA126-*AtACC2-BdMatB-BdMatC*	This study

Table S2. Primers used in this work

Primer	Sequence
STS-F	GGAGATCCGAAACGCCCAG
STS-R	TGACCATGGGAATAGAGTGCAGG
TAL-F	CCGACCTACCTCTCAGTCTCAG
TAL-R	AGCATCTTCAGCAGCACGTTG
4CL-F	CATGGAGAAGGACACCAAGCAG
4CL-R	CAGAATCTTGCCGGAGGGAG
ACC2-F	CCATTACCCTCACCAACATCTCC
ACC2-R	CTTAACAGTAGATGGTTCGGGTCTTGG
matB-F	GATGAACCGAGCCGCTAACG
matB-R	CTCGGAGCACGTTCTTCTGG
matC-F	CATTCTGAACCTCGCTCTGCC
matC-R	GAATGGCCCACATGACGGAG
STC-F	CTAAGGGACCCGCTACCATC
STC-R	GAATCTTGCCGGAGGGAGAC
BCA-F	CCAACCTCTTCTCCCGACTG
BCA-R	CGGGTCTTGGTAATGGTGGA

Table S3. The highest resveratrol yield among *Yarrowia lipolytica* and their engineering strategies

No	Host	Pathway Genes (Source)	Pathway/Host Engineering	Genetic System	Precursor	Titer (mg/L)	Scale	Reference
**1**	*Y. lipolytica* ATCC 20362	*RgPAL/TAL, Sc4CL VvSTS*	-	One copy genome integration	L-tyrosine	1.46		[3]
**2**	*Y. lipolytica*	*Nt4CL AhSTS*	Overexpression of: *ACC1, PEX10*	One copy genome integration	*p*-Coumaric acid	48.7	Flask	[4]
**3**	*Y. lipolytica* Po1d	*FjTAL, VvPAL, VvC4H, At4CL1, VvSTS*	-	Multiple copy genome integration	Glycerol	430	Bioreactor	[5]
**4**	*Y. lipolytica* Po1fk	*RtTAL, Pc4CL, VvVTS*	*ScARO4*^{fbr}, *EcaroG*^{fbr} (*E. coli*), *BbxfpK, AvxpkA* Overexpression: of *ARO1, ARO2, ARO3, ARO4, ARO5, TKT*. Deletion of: *TRP2,TRP3, ARO8, ARO9, PYK, PHA2*	One copy genome integration	Glucose	12.67	Flask	[6]
**5**	*Y. lipolytica* ST6512 (W29)	*FjTAL, At4CL1, VvVTS*	Overexpression of:*ARO4*^{fbr,} *ARO7*^{fbr}	Multiple copy genome integration	Glucose	12355	Fed-batch bioreactor	[7]
**6**	*Yarrowia lipolytica* Po1f	*FjTAL, Pc4CL1::VvSTS, SmPAL, SmC4H,*	*AtATR2, CaFPK, BsPTA* Overexpression of *YlARO4*^{K221L}, *YlARO7*^{G139S}, *YlARO1, YlCYB5, YlARO3*^{K225L} Knockout *DGA1*	Multiple copy genome integration	Glucose	22500	Fed-batch bioreactor	[8]

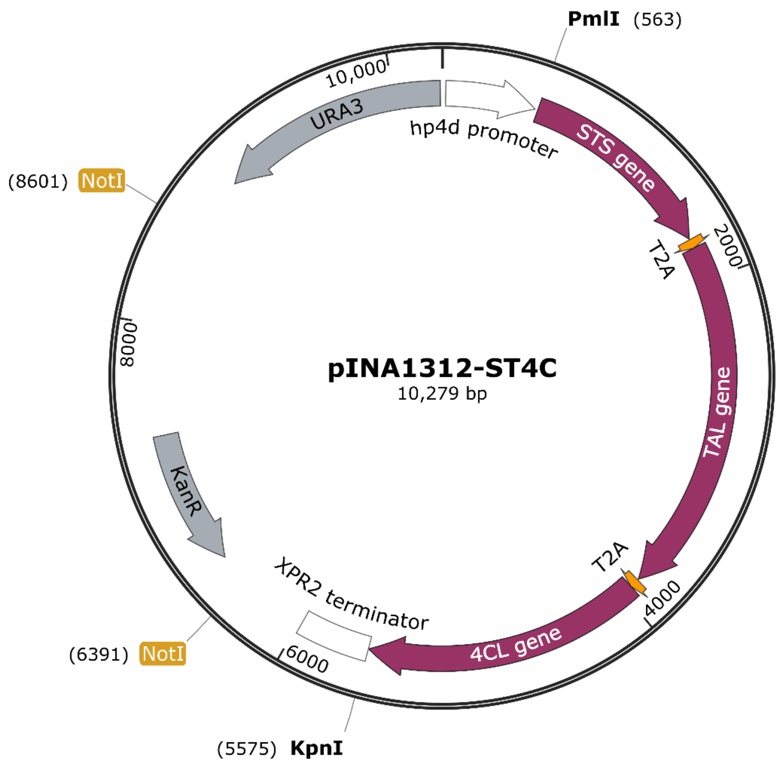

Figure S1. Schematic diagram of pINA1312-ST4C recombinant plasmid with *VvSTS, RgTAL*, and *Nt4CL* genes, linked by T2A linker.

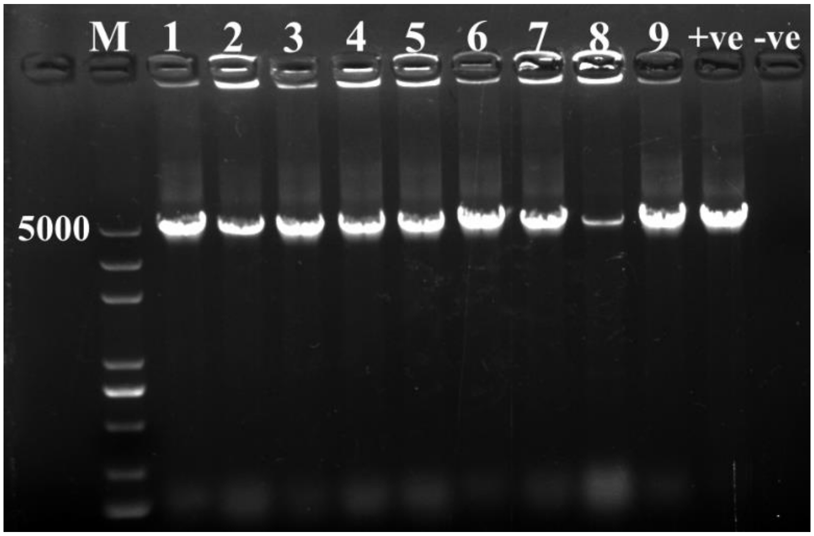

Figure S2. Colony PCR results of *E. coli* TOP10 recombinants with pINA1312-ST4C plasmid. M: DS 5000 marker; 1-9: recombinants DNA were used as a template; +ve: positive control where the pINA1312-ST4C plasmid was used as a DNA template; -ve: negative control where the reaction lacked a DNA template.

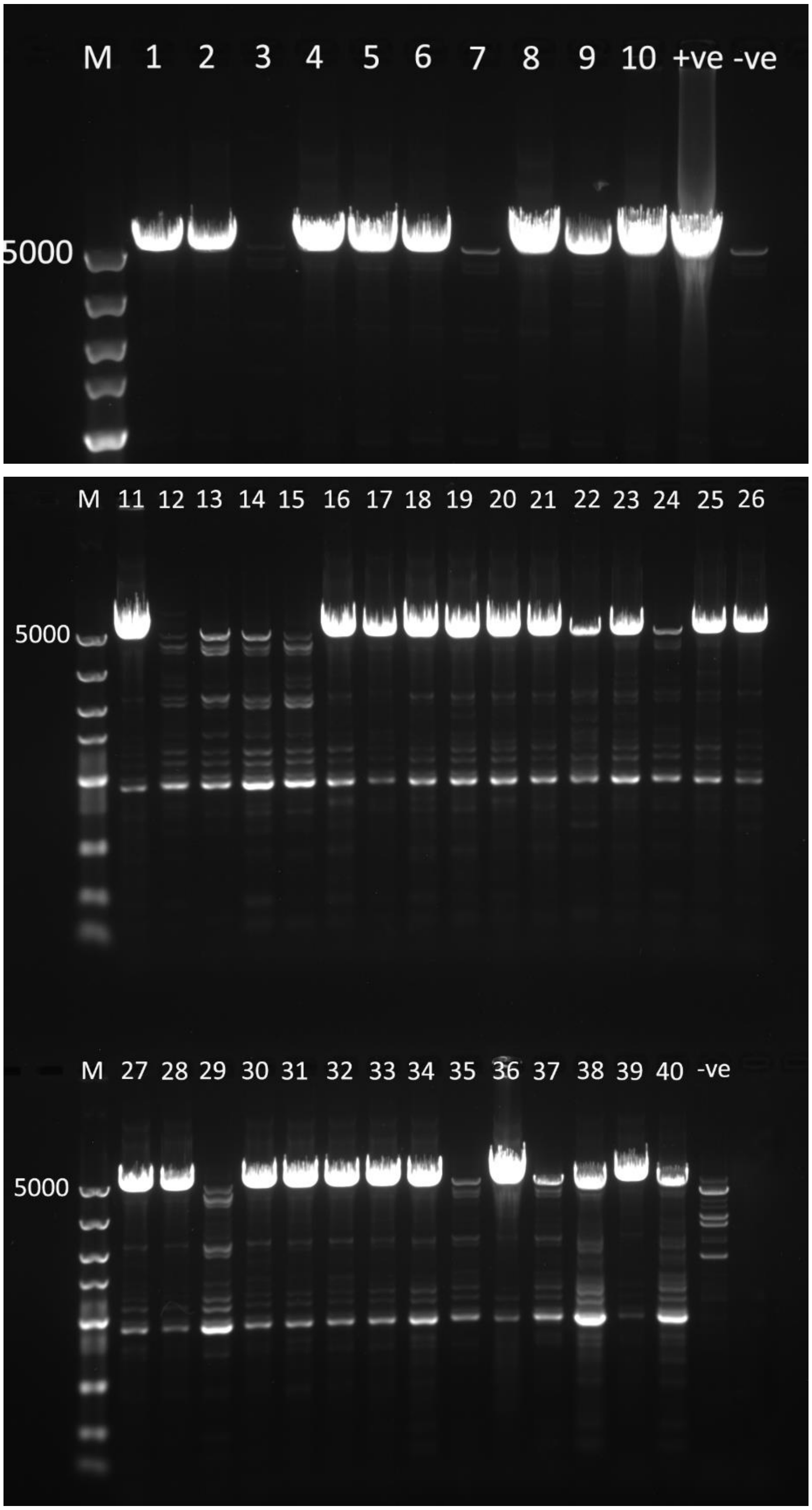

Figure S3. PCR results of *Y. lipolytica* Po1f recombinant strains with pINA1312-ST4C plasmid. M: DS 5000 marker; 1-40: recombinants DNA were used as a template; +ve: positive control where the plasmid DNA was used as a template; -ve: negative control where DNA extracted from the non-recombinant parental strain was used as a template.

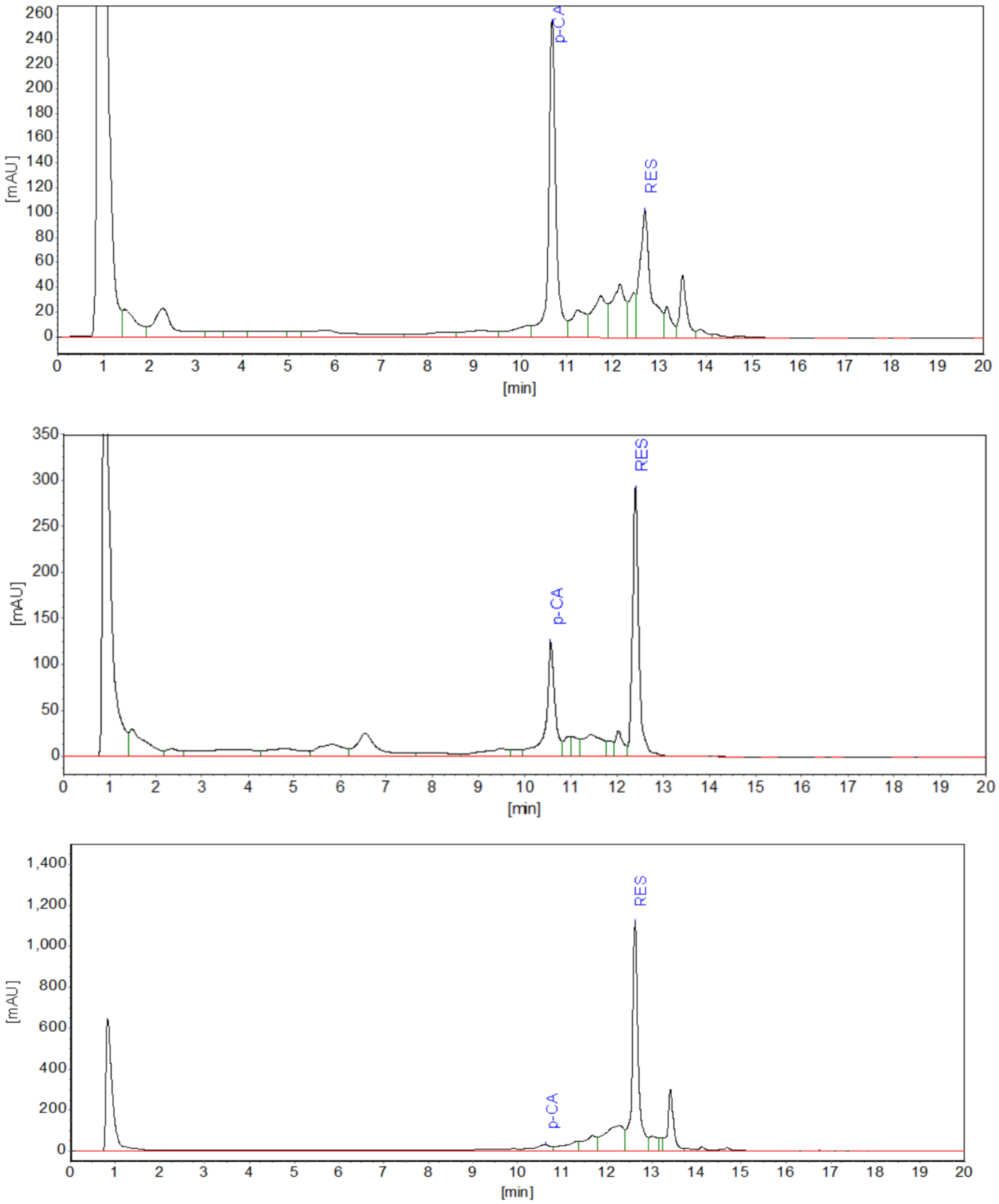

Figure S4. HPLC chromatographs for the *p*-CA and resveratrol peaks in selected samples. *p*-CA and resveratrol were extracted from YPD media, inoculated with different transformants, and fermented for 5 days. The *p*-CA and resveratrol peaks were detected at 306 nm.

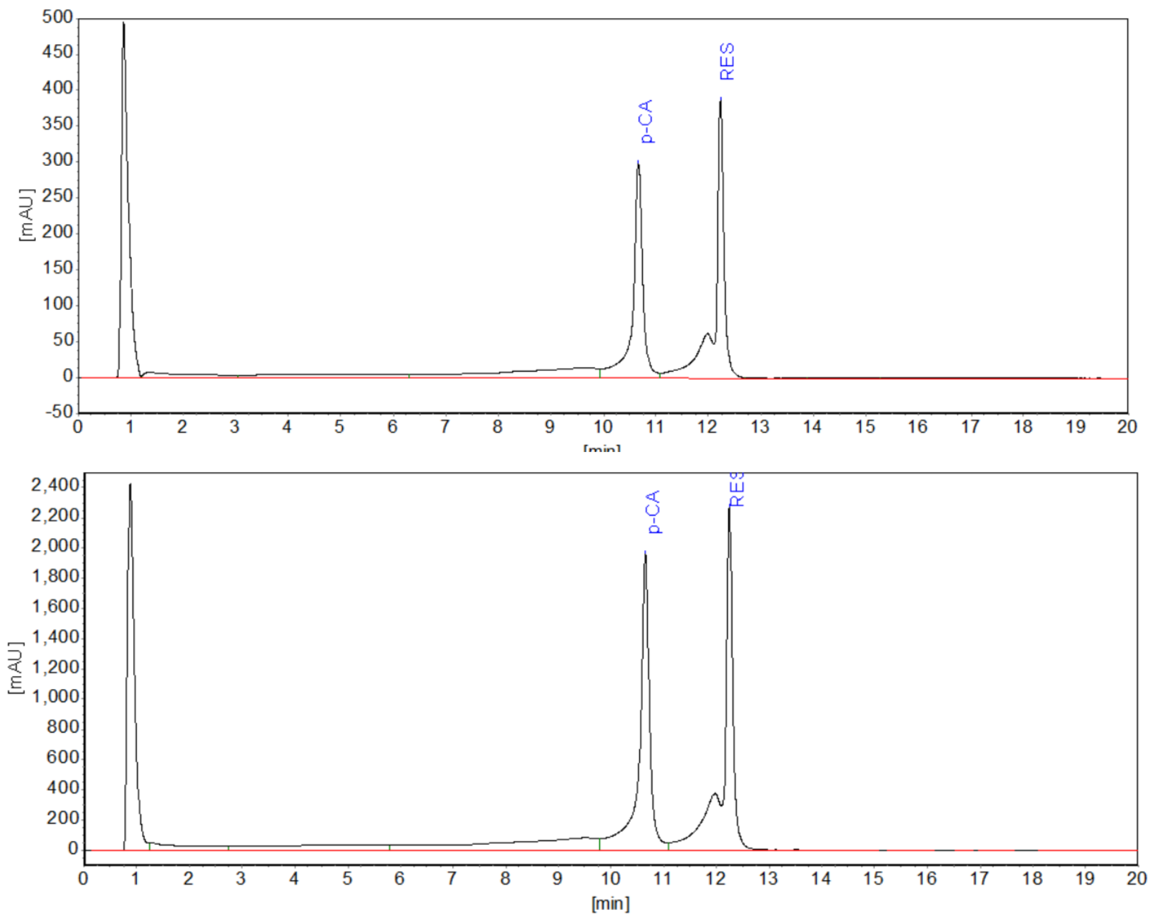

Figure S5. The HPLC chromatograph for *p*-CA and resveratrol standards. The chromatographs represent the peaks of 2 selected concentrations from standard *p*-CA and resveratrol. Both *p*-CA and resveratrol were detected at 306 nm.

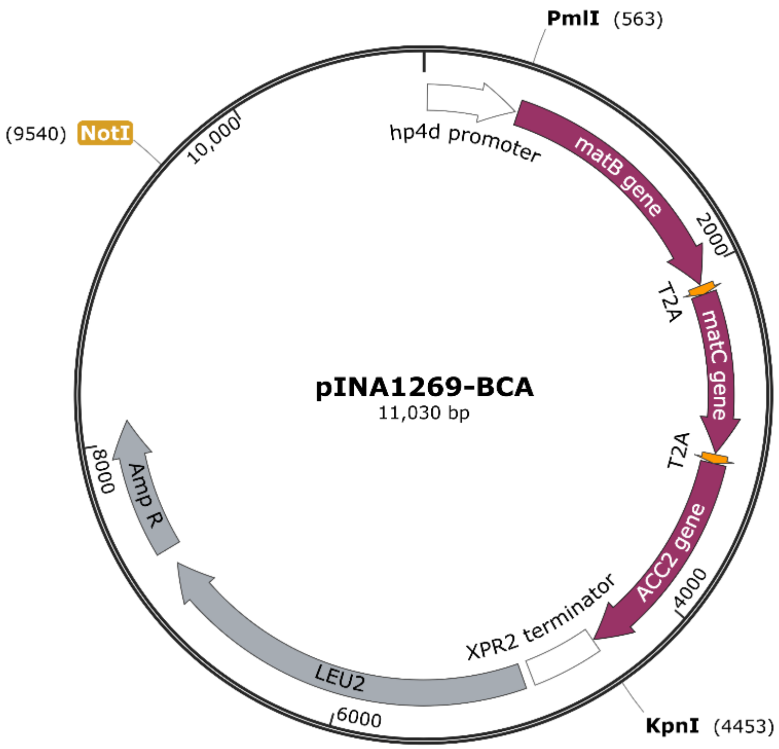

Figure S6. Schematic diagram of pINA1269-BCA recombinant plasmid containing *BdmatB, BdmatC,* and *AtACC2* genes, linked by T2A linker.

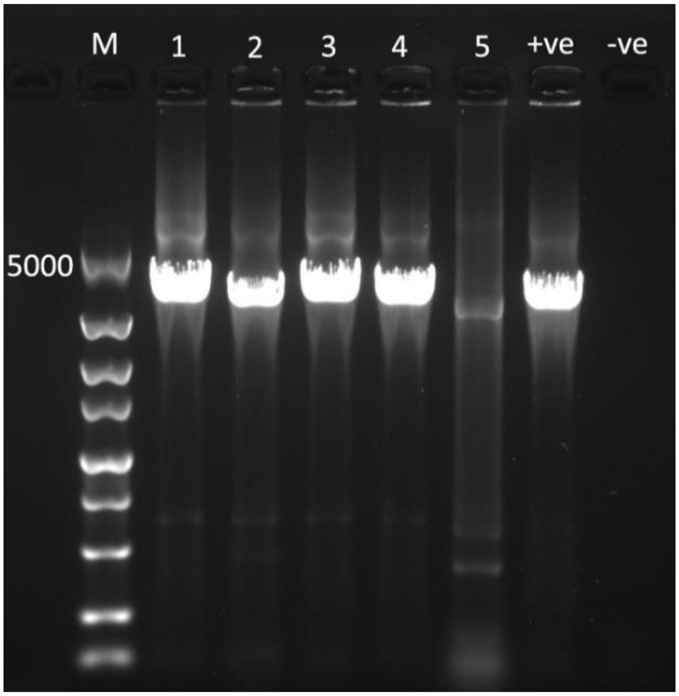

Figure S7. Colony PCR results of *E. coli* TOP10 recombinants with pINA1269-BCA plasmid. M: DS 5000 marker; 1-5: recombinants DNA were used as a template; +ve: positive control where the pINA1269-BCA plasmid was used as a DNA template; -ve: negative control where the reaction lacked a DNA template.

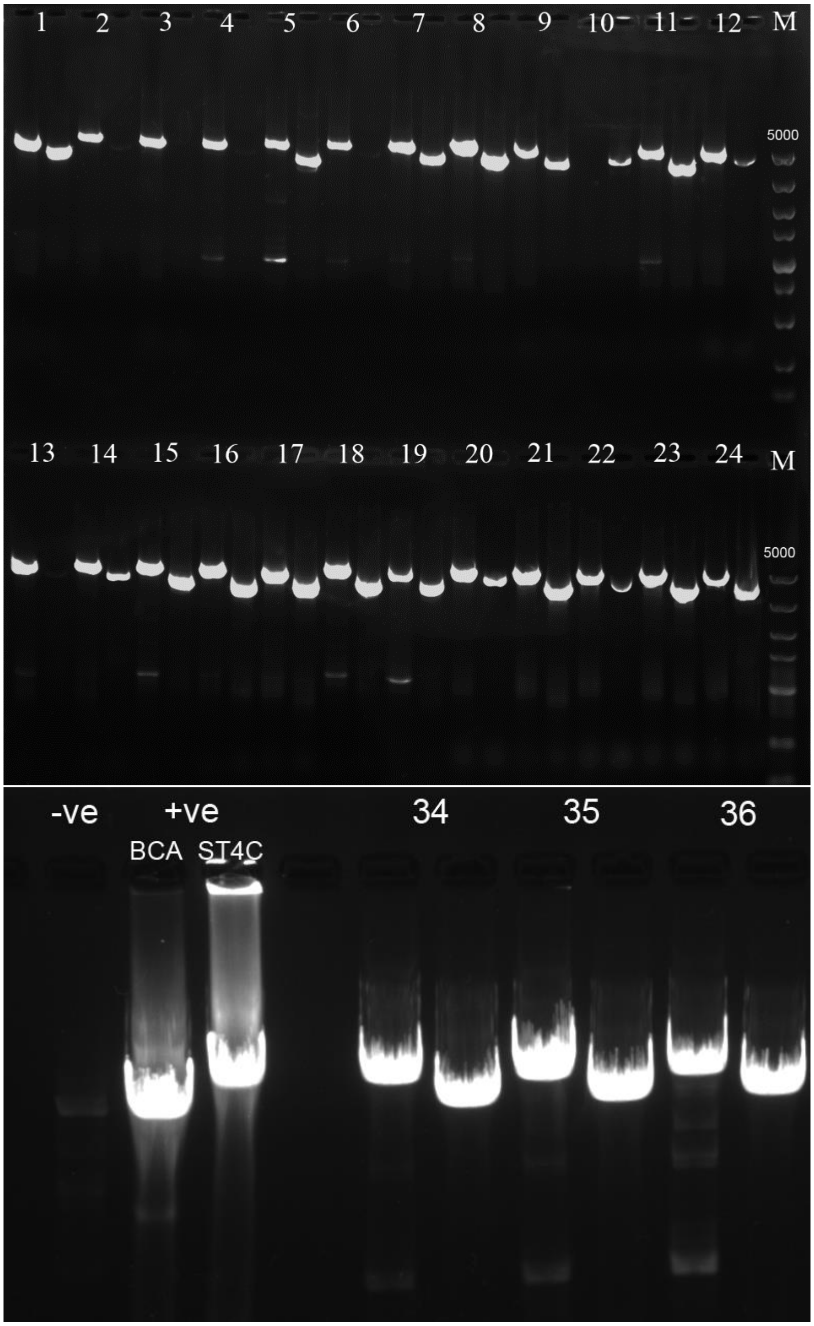

Figure S8. PCR confirmation results of transformant strains originating from S10. For each derived strain, dual-lane analysis was conducted to ascertain the presence of the plasmids, pINA1312-ST4C in the first lane and pINA1269-BCA in the second lane; M: DS 5000 marker; Lanes 1-36: recombinants DNA were used as templates; +ve (ST4C, BCA): positive controls where the plasmids pINA1312-ST4C and pINA1269-BCA were used as DNA template, respectively; -ve: negative control where the genomic DNA from the non-recombinant parental strain was used as template.

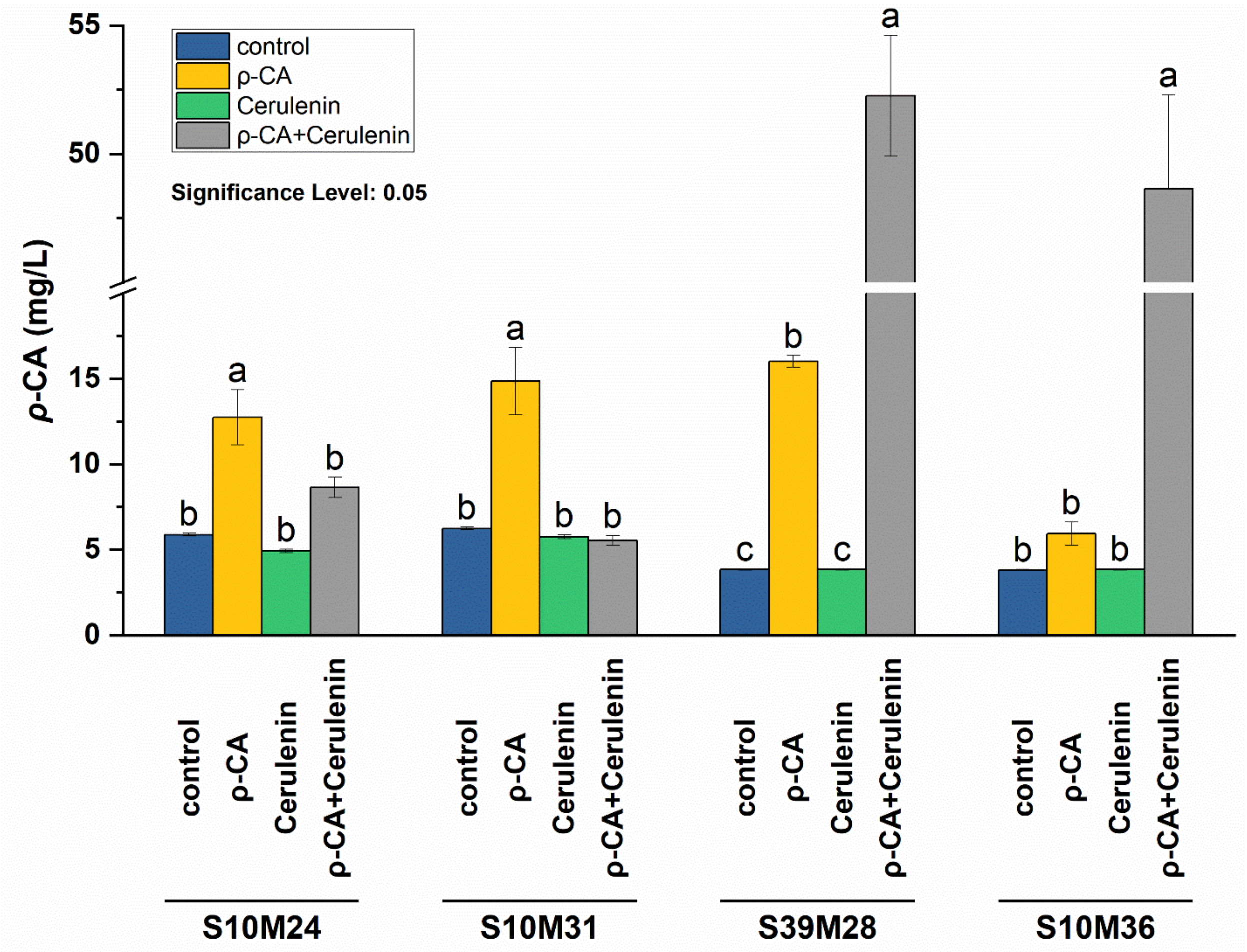

Figure S9. The effect of adding *p*-CA and cerulenin to the YNB media on *p*-CA production in different strains after 120 h culturing in YNB media. Letters represent the significance of differences. Distinct letters indicate significant differences at a 0.05 significance level.

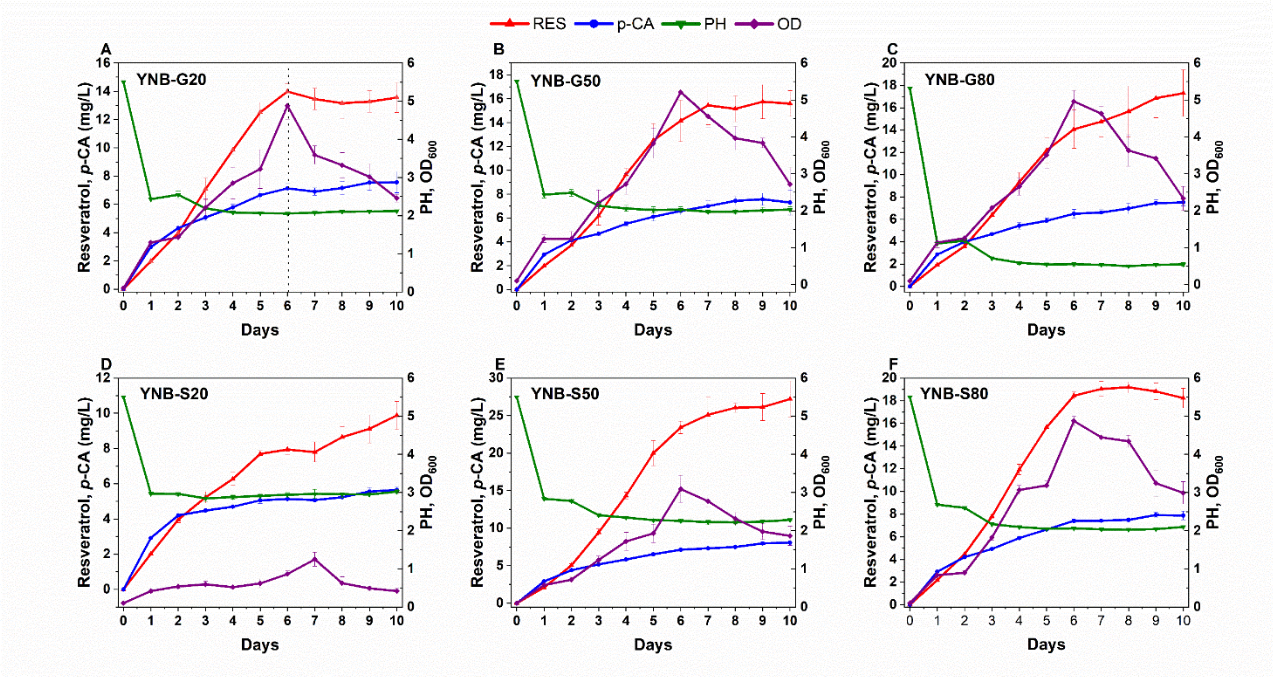

Figure S10. The effect of carbon source and its concentration on resveratrol production by S10M31 strain. The strain was fermented for 10 days in YNP media supplemented with 20, 50, or 80 g/L of glucose or sucrose as a carbon source. Dotted lines refer to the depletion of the carbon source.

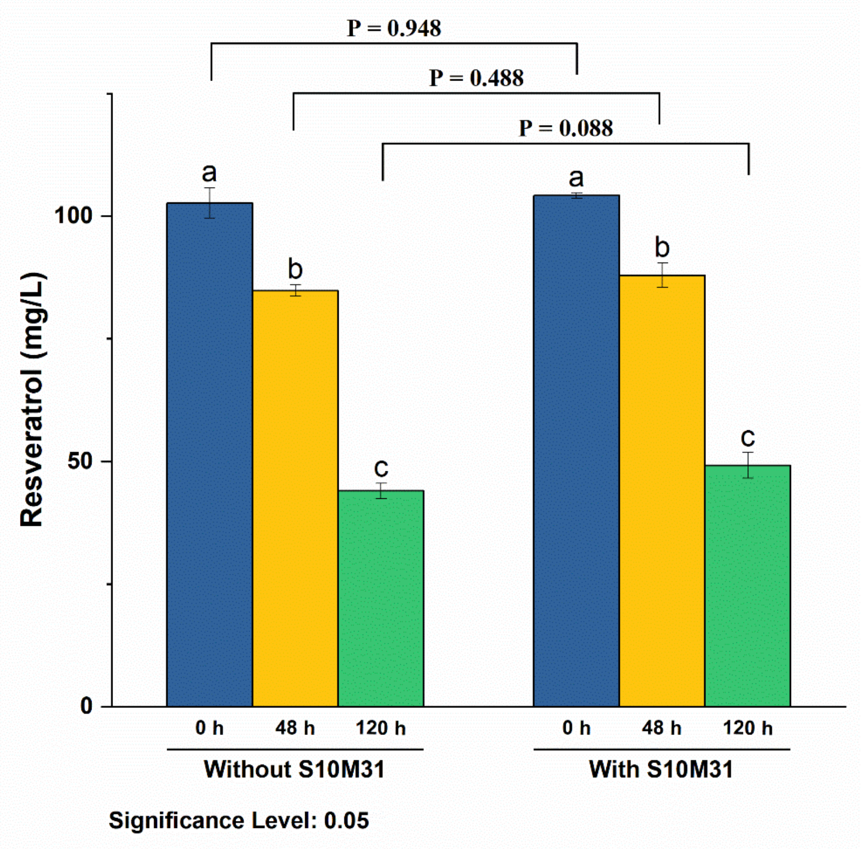

Figure S11. Degradation assays for resveratrol in S10M31 strain. A 100mg/L resveratrol was added as a single carbon source in YP media and incubated at 28 °C and 200 rpm for 120 h with or without inoculation with the S10M31 strain. The blue bars showed resveratrol concentration at the beginning of the experiment; the yellow bars showed resveratrol concentration after 48 h of incubation; the green bars showed resveratrol concentration after 120 h of incubation. Letters represent the significance of differences. Distinct letters indicate significant differences at a 0.05 significance level.

DNA sequences used in this work

Optimized Sequence STS-TAL-4CL

CACGTGATGGCCTCTGTGGAGGAGATCCGAAACGCCCAGCGAGCTAAGGGACCCGC
TACCATCCTGGCCATTGGAACCGCTACCCCCGACCACTGTGTCTACCAGTCCGACTA
CGCCGACTACTACTTCCGAGTGACCAAGTCTGAGCACATGTCCGAGCTGAAGAAGA
AGTTCAACCGAATTTGCGACAAGTCTATGATCAAGAAGCGATACATTCACCTGACCG
AGGAGATGCTGGAGGAGCACCCCAACATCGGAGCCTACATGGCTCCCTCCCTGAAC
ATTCGACAGGAGATCATTACCGCTGAGGTGCCCAAGCTGGGCAAGGAAGCCGCTCT
GAAGGCCCTGAAGGAGTGGGGCCAGCCCAAGTCTAAGATCACCCACCTGGTCTTCT
GTACCGCTTCTGGAGTGGAGATGCCTGGAGCTGACTACAAGCTGGCTAACCTGCTGG
GACTGGAGACCTCTGTGCGACGAGTCATGCTGTACCACCAGGGTTGTTACGCTGGCG
GAACCGTCCTGCGAACCGCCAAGGACCTGGCTGAGAACAACGCTGGAGCCCGAGTG
CTGGTGGTCTGCTCTGAGATTACCGTGGTCACCTTCCGAGGCCCCTCTGAGGACGCT
CTGGACTCTCTGGTGGGACAGGCTCTGTTCGGTGACGGTTCTGCTGCTGTGATCGTG
GGTTCTGACCCTGACGTCTCCATTGAGCGACCCCTGTTCCAGCTGGTGTCTGCCGCTC
AGACCTTCATTCCCAACTCCGCTGGAGCCATCGCTGGTAACCTGCGAGAAGTGGGAC
TGACCTTCCACCTGTGGCCCAACGTCCCCACCCTGATCTCCGAGAACGTGGAGAAGT
GTCTGACCCAGGCCTTCGACCCCCTGGGTATTTCTGACTGGAACTCCCTGTTCTGGAT
TGCTCACCCCGGTGGCCCCGCTATTCTGGACGCTGTGGAGGCTAAGCTGAACCTGGA
CAAGAAGAAGCTGGAGGCCACCCGACACGTCCTGTCTGAGTACGGAAACATGTCTT
CCGCTTGCGTGCTGTTCATCCTGGACGAGATGCGAAAGAAGTCCCACAAGGGAGAG
AAGGCTACCACCGGAGAGGGTCTGGACTGGGGTGTGCTGTTCGGCTTCGGACCCGG
TCTGACCATCGAGACCGTGGTCCTGCACTCTATTCCCATGGTCACCAACCGAGCTGA
GGGTCGAGGATCCCTGCTGACCTGTGGCGACGTGGAGGAGAACCCCGGACCCATGG
CTCCCCGACCTACCTCTCAGTCTCAGGCTCGAACCTGCCCTACCACCCAGGTGACCC
AGGTCGACATCGTGGAGAAGATGCTGGCTGCTCCTACCGACTCCACCCTGGAGCTG
GACGGTTACTCTCTGAACCTGGGCGACGTGGTCTCCGCTGCTCGAAAGGGTCGACCC
GTGCGAGTCAAGGACTCTGACGAGATCCGATCCAAGATTGACAAGTCTGTCGAGTTC
CTGCGATCTCAGCTGTCTATGTCCGTCTACGGCGTGACCACCGGATTCGGAGGTTCT

GCCGACACCCGAACCGAGGACGCTATCTCCCTGCAGAAGGCCCTGCTGGAGCACCA
GCTGTGTGGAGTGCTGCCCTCTTCCTTCGACTCTTTCCGACTGGGTCGAGGCCTGGA
GAACTCTCTGCCCCTGGAGGTGGTCCGAGGCGCCATGACCATTCGAGTCAACTCCCT
GACCCGAGGACACTCTGCTGTGCGACTGGTGGTCCTGGAGGCCCTGACCAACTTCCT
GAACCACGGCATCACCCCCATTGTCCCCCTGCGAGGAACCATCTCTGCCTCCGGTGA
CCTGTCTCCCCTGTCCTACATCGCCGCTGCCATTTCCGGTCACCCCGACTCTAAGGTG
CACGTCGTCCACGAGGGCAAGGAGAAGATTCTGTACGCCCGAGAGGCCATGGCTCT
GTTCAACCTGGAGCCCGTGGTCCTGGGACCCAAGGAAGGCCTGGGTCTGGTCAACG
GCACCGCCGTGTCTGCTTCTATGGCTACCCTGGCTCTGCACGACGCTCACATGCTGT
CTCTGCTGTCTCAGTCCCTGACCGCCATGACCGTCGAGGCTATGGTGGGTCACGCCG
GCTCCTTCCACCCCTTCCTGCACGACGTCACCCGACCCCACCCTACCCAGATTGAGG
TGGCTGGTAACATCCGAAAGCTGCTGGAGGGCTCTCGATTCGCCGTGCACCACGAG
GAAGAGGTGAAGGTCAAGGACGACGAGGGTATCCTGCGACAGGACCGATACCCCCT
GCGAACCTCTCCCCAGTGGCTGGGACCCCTGGTCTCCGACCTGATCCACGCCCACGC
TGTGCTGACCATTGAGGCCGGACAGTCTACCACCGACAACCCCCTGATCGACGTCGA
GAACAAGACCTCCCACCACGGCGGAAACTTCCAGGCTGCCGCTGTGGCTAACACCA
TGGAGAAGACCCGACTGGGTCTGGCCCAGATTGGCAAGCTGAACTTCACCCAGCTG
ACCGAGATGCTGAACGCCGGTATGAACCGAGGCCTGCCCTCTTGTCTGGCCGCTGAG
GACCCCTCTCTGTCCTACCACTGTAAGGGTCTGGACATTGCCGCTGCCGCTTACACC
TCTGAGCTGGGACACCTGGCTAACCCCGTGACCACCCACGTCCAGCCTGCTGAGATG
GCTAACCAGGCCGTCAACTCCCTGGCCCTGATTTCTGCTCGACGAACCACCGAGTCC
AACGACGTCCTGTCTCTGCTGCTGGCCACCCACCTGTACTGCGTGCTGCAGGCTATC
GACCTGCGAGCCATTGAGTTCGAGTTCAAGAAGCAGTTCGGACCCGCTATCGTGTCT
CTGATTGACCAGCACTTCGGATCCGCCATGACCGGTTCTAACCTGCGAGACGAGCTG
GTCGAGAAGGTGAACAAGACCCTGGCCAAGCGACTGGAGCAGACCAACTCTTACGA
CCTGGTCCCCCGATGGCACGACGCTTTCTCCTTCGCTGCTGGCACCGTGGTGGAGGT
GCTGTCTTCCACCTCTCTGTCCCTGGCCGCTGTCAACGCTTGGAAGGTGGCCGCTGC
CGAGTCTGCCATCTCCCTGACCCGACAGGTGCGAGAGACCTTCTGGTCCGCTGCTTC
CACCTCTTCCCCTGCTCTGTCTTACCTGTCTCCCCGAACCCAGATTCTGTACGCTTTC
GTCCGAGAGGAGCTCGGAGTGAAGGCCCGACGAGGCGACGTCTTCCTGGGAAAGCA
GGAAGTGACCATCGGTTCTAACGTGTCCAAGATCTACGAGGCCATTAAGTCTGGCCG

AATTAACAACGTGCTGCTGAAGATGCTGGCCCGAGCTGAGGGACGAGGCTCCCTGC
TGACCTGTGGTGACGTCGAAGAAAACCCCGGACCCATGGAGAAGGACACCAAGCAG
GTGGACATCATTTTCCGATCCAAGCTGCCCGACATCTACATTCCCAACCACCTGCCC
CTGCACTCCTACTGTTTCGAGAACATCTCTGAGTTCTCTTCCCGACCCTGCCTGATTA
ACGGCGCTAACAAGCAGATCTACACCTACGCCGACGTCGAGCTGAACTCTCGAAAG
GTGGCTGCCGGTCTGCACAAGCAGGGCATTCAGCCCAAGGACACCATCATGATTCT
GCTGCCCAACTCCCCCGAGTTCGTGTTCGCCTTCATCGGAGCTTCCTACCTGGGTGCT
ATTTCTACCATGGCTAACCCTCTGTTCACCCCTGCTGAGGTGGTCAAGCAGGCTAAG
GCTTCTTCCGCTAAGATCATTGTCACCCAGGCCTGCCACGTCAACAAGGTGAAGGAC
TACGCTTTCGAGAACGACGTGAAGATCATTTGTATCGACTCTGCCCCCGAGGGATGC
CTGCACTTCTCCGTCCTGACCCAGGCTAACGAGCACGACATCCCCGAGGTGGAGATT
CAGCCTGACGACGTGGTGGCTCTGCCTTACTCTTCCGGAACCACCGGTCTGCCCAAG
GGAGTCATGCTGACCCACAAGGGTCTGGTCACCTCTGTGGCTCAGCAGGTGGACGG
CGAGAACCCCAACCTGTACATCCACTCCGAGGACGTCATGCTGTGTGTGCTGCCCCT
GTTCCACATCTACTCTCTGAACTCCGTCCTGCTGTGCGGACTGCGAGTGGGTGCTGC
CATCCTGATTATGCAGAAGTTCGACATTGTCTCTTTCCTGGAGCTGATCCAGCGATA
CAAGGTGACCATTGGACCCTTCGTGCCTCCCATCGTGCTGGCCATTGCTAAGTCTCC
CATGGTGGACGACTACGACCTGTCTTCCGTCCGAACCGTGATGTCTGGAGCTGCTCC
CCTGGGAAAGGAGCTGGAGGACACCGTCCGAGCCAAGTTCCCCAACGCCAAGCTGG
GTCAGGGCTACGGAATGACCGAGGCTGGTCCCGTGCTGGCCATGTGTCTGGCCTTCG
CTAAGGAGCCCTTCGAGATCAAGTCCGGCGCTTGCGGAACCGTGGTCCGAAACGCC
GAGATGAAGATTGTGGACCCTAAGACCGGTAACTCCCTGCCTCGAAACCAGTCTGG
CGAGATCTGCATTCGAGGAGACCAGATCATGAAGGGTTACCTGAACGACCCCGAGG
CCACCGCTCGAACCATTGACAAGGAAGGCTGGCTGTACACCGGTGACATCGGCTAC
ATTGACGACGACGACGAGCTGTTCATCGTCGACCGACTGAAGGAGCTGATTAAGTA
CAAGGGATTCCAGGTGGCTCCTGCTGAGCTGGAGGCTCTGCTGCTGAACCACCCCAA
CATCTCTGACGCTGCCGTGGTCCCCATGAAGGACGAGCAGGCTGGAGAGGTGCCCG
TCGCCTTCGTGGTCCGATCTAACGGTTCCACCATTACCGAGGACGAGGTCAAGGACT
TCATCTCCAAGCAGGTCATCTTCTACAAGCGAATCAAGCGAGTCTTCTTCGTGGACG
CCATCCCCAAGTCTCCCTCCGGCAAGATTCTGCGAAAGGACCTGCGAGCTAAGCTGG
CTGCCGGACTGCCCAACTAGGGTACC

Optimized Sequence matB-matC-ACC2

CACGTGATGAACCGAGCCGCTAACGCCAACCTCTTCTCCCGACTGTTCGACGGTCTC
GACGACCCCAAGCGACTGGCTATCGAGACCCACGACGGCGCCCGAATTTCTTACGG
TGACCTGATCGCTCGAGCCGGCCAGATGGCCAACGTCCTCGTGGCTCGAGGTGTCAA
GCCTGGAGACCGAGTGGCTGTGCAGGTCGAGAAGTCCGTCGCTAACATTGTGCTGTA
CCTCGCCACCGTCCGAGCTGGTGCTGTGTACCTGCCTCTCAACACCGCTTACACCCT
CAACGAGCTGGACTACTTCATTGGAGACGCTGAGCCTTCCCTGGTGGTTTGTGACCC
TTCTAAGGCTGAGGGACTGGCTCCCATTGCCGCTAAGGTCAAGGCTGGAGTGGAGA
CCCTGGGTCCCGACGGAAAGGGCTCTCTCACCGAGGCCGCTGACAAGGCCTCCTCTG
CTTTCACCACCGTCCCCCGAGAGAACGACGACCTGGCTGCTATCCTCTACACCTCTG
GAACCACCGGCCGATCCAAGGGTGCTATGCTGACCCACGACAACCTCGCCTCCAAC
TCTCTGTCCCTCGTCGGATACTGGCGATTCACCGACAAGGACGTGCTGATCCACGCC
CTCCCCATCTACCACACCCACGGCCTGTTCGTCGCTACCAACGTGACCCTCTTCTCTC
GAGCCTCCATGATCTTCCTCCCCAAGCTGGACCCCGACCTGATCATTAAGCTCATGG
CTCGAGCCACCGTCCTGATGGGTGTGCCCACCTTCTACACCCGACTGCTCCAGAACG
CCGCTCTGTCCCGAGAGACCACCCGACACATGCGACTCTTCATTTCTGGTTCTGCTCC
CCTGCTGGCTGAGACCCACCGAGAGTGGTCTGCCCGAACCGGTCACGCTGTCCTGGA
GCGATACGGAATGACCGAGACCAACATGAACACCTCCAACCCTTACGACGGAGAGC
GAGTCCCCGGTGCTGTGGGATTCCCTCTGCCTGGAGTCTCTCTCCGAGTGACCGACC
CTGAGACCGGCAAGGAGCTGCCCCGAGAGGAGATCGGAATGATTGAGGTCAAGGGT
CCCAACGTGTTCAAGGGTTACTGGCGAATGCCCGAGAAGACCAAGGCCGAGTTCCG
ACCCGACGGCTTCTTCATTACCGGCGACCTGGGCAAGATCGACGGCAAGGGATACG
TGCACATTCTGGGCCGAGGCAAGGACCTCGTCATCTCCGGCGGTTTCAACGTGTACC
CCAAGGAGATCGAGTCCGAGATTGACGCTATGCCCGGCGTCGTGGAGTCTGCTGTG
ATCGGTGTCCCCCACGCTGACTTCGGAGAGGGTGTCACCGCTGTGCTGGTCTGCAAC
AAGGGTGCCGAGGTCTCTGAGGCTTCCGTGCTCAAGGCCCTGGACGGACGACTCGC
CAAGTTCAAGATGCCCAAGCGAGTGTTCGTCGTGGACGAGCTGCCCCGAAACACCA
TGGGCAAGGTCCAGAAGAACGTGCTCCGAGACACCTACAAGGACATCTACGCCAAG
AAGCGAGCTGAGGGTCGAGGATCCCTGCTCACCTGCGGCGACGTGGAGGAGAACCC

CGGTCCCATGGTGGACATTCTGAACCTCGCTCTGCCCTACTTCGGACTGATTTTCGTC
GGCTTCGCCTGTGGCAAGATCAAGTCTCTGCCCGAGTCCGGCCTCGCCTGGATGAAC
TTCTTCCTGCTCTACGTGTCCCTGCCCGCCCTGCTCTTCGCTATCATGTCTAAGACCC
CCTTCGCTGAGCTCAACAACCCTCCCTTCCTGGTGGCTACCACCCTCTCCACCGTGAC
CGCCTTCACCCTCGCTCTGGTCGTGGGAAAGGTGCTCGGACGACTCACCCTGCGAGA
GGCTACCCTCGCTGGTCTGTCTGGAGGCTACGGAAACATCGGCTACATGGGTCCCGG
TCTGGCTCTGGCTGTGCTCGGAGCTAAGGCTTCTGCTCCTACCGCTCTGATTTTCTGC
TGTGACTCTATCTTCCTCTTCACCATTGTGCCCCTGCTCATCGAGCTGTCTGACCGAG
ACCACCCCTCCATCGTCCACGCCTTCGGTGTCGTGCTCAAGCAGATTGTGCTCAACC
CCCTGATCATGTCCGCTTGTTTCGGAGCCGCTGTCGCCGCTCTGCACATTGAGCTCCC
CGTGGCCCTGGACCGAACCATCACCTTCCTGCAGAACGCTGCTGCTCCCACCGCTCT
GTTCGTCCTGGGTGTGACCGTGGCTCTCCGACCTTTCGACCGAGTCCCCTGGGAGGT
GCCCGGTGTGGTGGCTGTGAAGCTGCTCTTCCACCCCCTGGCTGCCTTCGGACTCAT
GCTGGCTTTCGGTCCCTTCGCTCAGCCTTGGGCTGCTACCGCTGTCCTCATGGCTTCC
CTGCCTCCCGCCCTCAACGTGTTCGTCATTGCTCGACAGAACGACGCCTGGATCGAG
TCTGCCTCCGTGGCTGTCCTGCTCGGCACCTTCGCTTCTGTCGTGACCCTGACCTCCG
TCATGTGGGCCATTCAGACCGGTCGACTGGCTTTCCCCCGAGCTGAGGGACGAGGAT
CTCTGCTCACCTGCGGAGACGTCGAAGAAAACCCCGGTCCCATGGAGATGCGAGCT
CTGGGTTCCTCTTGTTCTACCGGAAACGGTGGATCCGCCCCCATTACCCTCACCAAC
ATCTCCCCCTGGATTACCACCGTCTTCCCCTCTACCGTGAAGCTGCGATCCTCTCTCC
GAACCTTCAAGGGCGTCTCCTCTCGAGTGCGAACCTTCAAGGGTGTCTCCTCTACCC
GAGTGCTGTCCCGAACCAAGCAGCAGTTCCCCCTGTTCTGCTTCCTCAACCCCGACC
CCATCTCCTTCCTGGAGAACGACGTCTCTGAGGCTGAGCGAACCGTGGTGCTCCCTG
ACGGTTCTGTCAACGGTGCCGGATCCGTGAACGGATACCACTCTGACGTCGTGCCCG
GCCGAAACGTGGCTGAGGTCAACGAGTTCTGTAAGGCTCTGGGCGGCAAGCGACCT
ATCCACTCTATTCTCGTGGCCACCAACGGAATGGCTGCCGTCAAGTTCATTCGATCC
GTGCGAACCTGGGCTTACGAGACCTTCGGCTCTGAGAAGGCCGTGAAGCTGGTCGC
CATGGCTACCCCCGAGGACATGCGAATCAACGCTGAGCACATCCGAATTGCTGACC
AGTTCGTGGAGGTCCCCGGAGGCACCAACAACAACAACTACGCCAACGTCCAGCTG
ATTGTGGAGATGGCTGAGGTGACCCGAGTCGACGCTGTGTGGCCCGGATGGGGTCA
CGCTTCTGAGAACCCTGAGCTGCCTGACGCTCTCAAGGAGAAGGGTATCATCTTCCT

GGGTCCTCCCGCTGACTCTATGATCGCCCTGGGTGACAAGATTGGATCCTCTCTCATT
GCTCAGGCTGCTGACGTCCCTACCCTGCCCTGGTCTGGTTCCCACGTCAAGATCCCT
CCCGGACGATCCCTGGTGACCGTCCCCGAGGAGATCTACAAGAAGGCCTGCGTCTA
CACCACCGAGGAAGCCATTGCTTCCTGTCAGGTCGTGGGCTACCCCGCCATGATTAA
GGCTTCTTGGGGCGGCGGCGGCAAGGGTATCCGAAAGGTCCACAACGACGACGAGG
TGCGAGCCCTGTTCAAGCAGGTCCAGGGTGAGGTGCCCGGTTCTCCCATCTTCATTA
TGAAGGTCGCTTCTCAGTCTCGACACCTGGAGGCTCAGCTGCTCTGCGACCAGTACG
GCAACGTGGCTGCCCTCCACTCTCGAGACTGCTCCGTCCAGCGACGACACCAGAAG
GTCTGTTACTGGTTCAAGATCGACCACATTTCTTTCTACTACTCCACCATTACCAAGA
CCCGAACCATCTACTGTTAAGGTACC

Reference

1. Madzak, C.; Tréton, B.; Blanchin-Roland, S. Strong hybrid promoters and integrative expression/secretion vectors for quasi-constitutive expression of heterologous proteins in the yeast *Yarrowia lipolytica*. *J. Mol. Microbiol. Biotechnol.* **2000**, *2*, 207–216.
2. Nicaud, J.-M.; Madzak, C.; van den Broek, P.; Gysler, C.; Duboc, P.; Niederberger, P.; Gaillardin, C. Protein expression and secretion in the yeast *Yarrowia lipolytica*. *FEMS Yeast Res.* **2002**, *2*, 371–379. https://doi.org/10.1016/S1567-1356(02)00082-X.
3. Huang, L.L.; Xue, Z.; Zhu, Q.Q. Method for the production of resveratrol in a recombinant oleaginous microorganism. United States E. I. du Pont de Nemours and Company, Wilmington, DE (US) 2010.
4. Palmer, C.M.; Miller, K.K.; Nguyen, A.; Alper, H.S. Engineering 4-coumaroyl-CoA derived polyketide production in *Yarrowia lipolytica* through a β-oxidation mediated strategy. *Metab. Eng.* **2020**, *57*, 174–181. https://doi.org/10.1016/j.ymben.2019.11.006.
5. He, Q.; Szczepańska, P.; Yuzbashev, T.; Lazar, Z.; Ledesma-Amaro, R. De novo production of resveratrol from glycerol by engineering different metabolic pathways in Yarrowia lipolytica. **2020**, 11e00146.
6. Gu, Y.; Ma, J.; Zhu, Y.; Ding, X.; Xu, P. Engineering *Yarrowia lipolytica* as a chassis for de novo synthesis of five aromatic-derived natural products and chemicals. *ACS Synth. Biol.* **2020**, *9*, 2096–2106. https://doi.org/10.1021/acssynbio.0c00185.
7. Sáez-Sáez, J.; Wang, G.; Marella, E.R.; Sudarsan, S.; Cernuda Pastor, M.; Borodina, I. Engineering the oleaginous yeast *Yarrowia lipolytica* for high-level resveratrol production. *Metab. Eng.* **2020**, *62*, 51–61. https://doi.org/10.1016/j.ymben.2020.08.009.
8. Liu, M.; Wang, C.; Ren, X.; Gao, S.; Yu, S.; Zhou, J. Remodelling metabolism for high-level resveratrol production in *Yarrowia lipolytica*. *Bioresour. Technol.* **2022**, *365*, 128178. https://doi.org/10.1016/j.biortech.2022.128178.
